# Fisetin Inhibited Growth and Metastasis of Triple-Negative Breast Cancer by Reversing Epithelial-to-Mesenchymal Transition via PTEN/Akt/GSK3β Signal Pathway

**DOI:** 10.3389/fphar.2018.00772

**Published:** 2018-07-31

**Authors:** Jie Li, Xia Gong, Rong Jiang, Dan Lin, Tao Zhou, Aijie Zhang, Hongzhong Li, Xiang Zhang, Jingyuan Wan, Ge Kuang, Hongyuan Li

**Affiliations:** ^1^Department of Endocrine and Breast Surgery, The First Affiliated Hospital of Chongqing Medical University, Chongqing, China; ^2^Molecular Oncology and Epigenetics Laboratory, The First Affiliated Hospital of Chongqing Medical University, Chongqing, China; ^3^Chongqing Key Laboratory of Biochemistry and Molecular Pharmacology, Chongqing Medical University, Chongqing, China; ^4^Department of Anatomy, Chongqing Medical University, Chongqing, China; ^5^Laboratory of Stem Cell and Tissue Engineering, Chongqing Medical University, Chongqing, China

**Keywords:** fisetin, triple negative breast cancer, EMT, PTEN, AKT

## Abstract

Triple negative breast cancer (TNBC), characterized by its highly aggressive and metastatic features, is associated with poor prognosis and high mortality partly due to lack of effective treatment. Fisetin, a natural flavonoid compound, has been demonstrated to possess anti-cancer effects in various cancers. However, the effects and mechanisms of fisetin on metastasis of TNBC remain uncovered. In this study, we found that fisetin dose-dependently inhibited cell proliferation, migration and invasion in TNBC cell lines MDA-MB-231 and BT549 cells. In addition, fisetin reversed epithelial to mesenchymal transition (EMT) as evaluated by cell morphology and EMT markers in MDA-MB-231 and BT549 cells. Furthermore, fisetin suppressed phosphoinositol 3-kinase (PI3K)-Akt-GSK-3β signaling pathway but upregulated the expression of PTEN mRNA and protein in a concentration-dependent manner. Further, silence of PTEN by siRNA abolished these benefits of fisetin on proliferation and metastasis of TNBCs. *In vivo*, using the metastatic breast cancer xenograft model bearing MDA-MB-231 cells, we found that fisetin dramatically inhibited growth of primary breast tumor and reduced lung metastasis, meanwhile, the expression of EMT molecules and PTEN/Akt/GSK-3β in primary and metastatic tissues changed in the same way as those *in vitro* experiments. In conclusion, all these results indicated that fisetin could effectively suppress proliferation and metastasis of TNBC and reverse EMT process, which might be mediated by PTEN/Akt/GSK-3β signaling pathway.

## Introduction

Breast cancer is the most common malignant tumor in women, and it is the most important reason causing cancer death among women, either in developed countries or developing countries. In 2012, there were 1,383,500 women who were diagnosed with breast cancer all over the world, and about 458,400 of them died because of breast cancer (Torre et al., 2015). Although plenty of drugs and synthetic treatment strategies have been utilized extensively in clinic, the incidence rate of treatment failure and cancer recurrence still remains at a high level, overwhelmingly because of cancer metastasis (Wculek and Malanchi, 2015). Thus, it can be seen that metastatic breast cancer is an increasing threat for global women’s health. To explore the mechanisms of metastasis and find the new drugs to aim directly at it may provide a better prognosis for patients.

Epithelial to mesenchymal transition (EMT) is a common phenotypic conversion involved in both normal physiological and pathological processes including embryonic development, wounded tissues plerosis, and the tumor metastasis and dissemination (Takebe et al., 2011; Nieto et al., 2016). EMT makes it possible for cancer cells to detach from the primary tumor site and encroach into the surrounding normal tissues, lymphatic and blood system, where they are disseminated to distant sites to form the metastatic lesion (Nickel and Stadler, 2015). During the EMT process of tumor metastasis, the cancer cells complete the shift from the adhesive, non-mobile, oval epithelial phenotype to the mobile, invasive long spindle mesenchymal phenotype, meanwhile, the epithelial cell markers like E-cadherin and Claudin were down-regulated but the mesenchymal cell markers such as N-cadherin and Vimentin were up-regulated (Banyard and Bielenberg, 2015). There are plenty of transcriptional factors like Slug, Snail, ZEB, Twist and signaling molecules such as Wnt, Notch, TGF-β, and ErbB to be involved in the regulation of EMT (Takebe et al., 2011; Moyret-Lalle et al., 2014). Besides cancer metastasis, a series of researches have confirmed that EMT also has connection with emergence of drug resistance and acquisition of cancer stem cells feature, which make the treatment of cancer sink into a more difficult situation (Huang et al., 2015).

**FIGURE 1:**
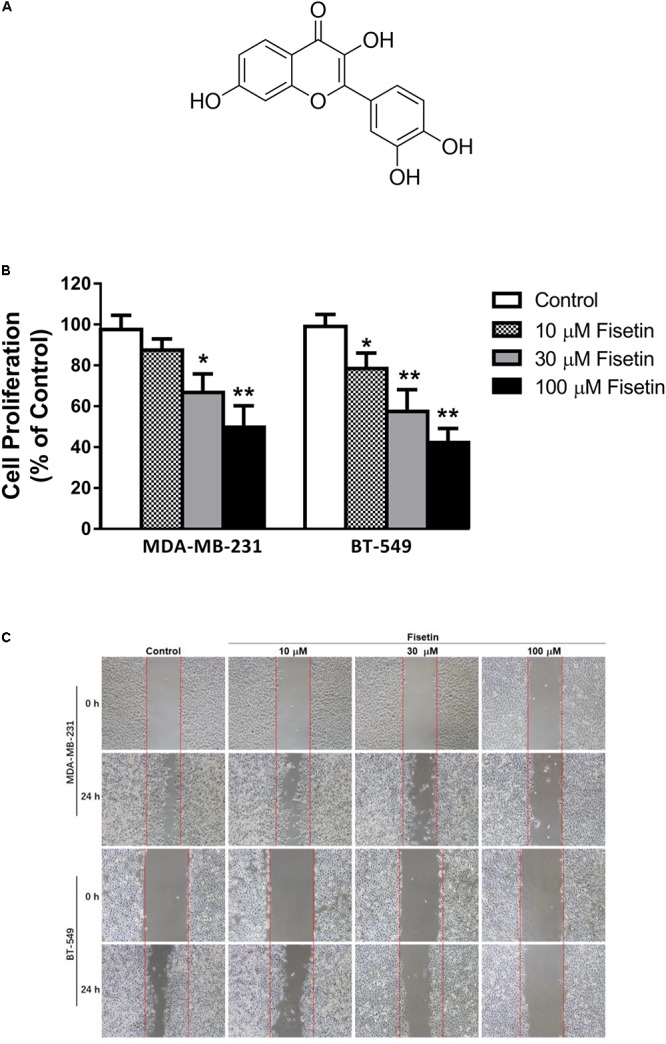
Fisetin suppresses the proliferation, migration and invasion of TNBC cells *in vitro*. Triple-negative breast cancer (TNBC) cell lines MDA-MB-231 and BT549 were treated with various concentrations of fisetin (10, 30, and 100 μM) for indicated time. **(A)** Chemical structure of fisetin. **(B)** The cell proliferation was determined by MTT assay at 72 h after fisetin treatment. **(C)** The cell migration was determined by wound-healing assay. **(D)** Quantification of the migrated cells. **(E)** The cell invasion was determined by transwell invasion assay. **(F)** Quantification of the invasive cells. The results are shown as the mean ± SD of three experiments, ^∗^*P* < 0.05, ^∗∗^*P* < 0.01 compared with control.

Along with the continual incidence of drug resistance and the serious adverse effects of the routine agents for chemotherapy in the treatment of cancers, more and more studies transfer their focus on the potential of natural plant compounds (Rasool et al., 2015). Fisetin (3,3′,4′,7-tetrahydroxyflavone, **Figure 1A**) is one of the major flavonoids that can be extracted from many fruits and vegetables like strawberry, apple, persimmon, grape, onion, and cucumber (Khan et al., 2013). It has been reported that fisetin exerted a series of pharmacological functions including anti-oxidant, anti-inflammatory, anti-angiogenesis, and anti-tumor (Bhat et al., 2012; Pal et al., 2016; Rengarajan and Yaacob, 2016; Sechi et al., 2016). In addition, several researches have shown that fisetin exhibited dramatically inhibitory effects on tumor progression by suppressing cancer cell growth, migration, invasion, and autophagy process and promoting cell cycle arrest and apoptosis in various of cancers, such as nasopharyngeal carcinoma, melanoma, lung cancer, breast cancer, bladder cancer, hepatocellular carcinoma, and prostate cancer (Li et al., 2011, 2014; Khan et al., 2012, 2014; Yang et al., 2012; Syed et al., 2014; Kang et al., 2015; Maurya and Trigun, 2017). However, the effects and underlying mechanisms of fisetin on the growth and metastasis of triple-negative breast cancer (TNBC) still remain unclear.

In this study, we chose two fibroblastic human TNBC cell lines MDA-MA-231 and BT549, which are utilized extensively as highly aggressive breast cancer cell lines (Lehmann and Pietenpol, 2014), to investigate the potential effects and mechanisms of fisetin on the growth and metastasis of TNBC *in vitro* and *in vivo*.

## Materials and Methods

### Reagents and Antibodies

Fisetin (purity > 99%) was bought from Push Bio-Technology (Chengdu, China). 3-(4,5 dimethylthiazol-2-yl)-2,5-diphenyltetrazolium bromide (MTT) was purchased from Sigma (St. Louis, United States). Vectastain ABC kit was obtained from Vector (Burlingame, CA, United States). The liquid DAB+ Substrate Chromogen System was purchased from Dako (California, United States). Dulbecco’s Modified Eagle’s (DMED) medium, trypsin-EDTA, phosphate buffer saline (PBS) and penicillin/streptomycin were bought from Hyclone (Los Angeles, CA, United States). Fetal bovine serum (FBS) was the product of Gibco (Grand Island, NY, United States). RNA extraction, PrimeScript RT and PCR reagent kits were bought from TaKaRa (Dalian, China). Primary antibodies used in this study including anti-Vimentin, anti-E-cadherin, anti-Claudin, anti-N-cadherin, anti-Slug, anti-Snail, anti-PTEN, anti-p-Akt, anti-Akt, anti-p-GSK-3β were obtained from Cell Signaling Technology (New England Biolabs, Ipswich, MA, United States), anti-Ki67 was from Abcam (Cambridge, United Kingdom). Secondary antibody (rabbit monoclonal IgG) was from Abcam (Cambridge, United Kingdom). Anti-GAPDH was from Santa Cruz Biotechnology (Santa Cruz, CA, United States). Anti-F-actin-Red 555 was from Invitrogen (Carlsbad, CA, United States).

### Cell Culture and Treatment

Human TNBC cell lines MDA-MB-231 and BT549 were obtained from the American Type Culture Collection (ATCC) (Manassas, VA, United States) and cultured in Dulbecco’s Modified Eagle Media (DMEM) medium with 10% fetal bovine serum (FBS) and 1% penicillin/streptomycin in a humidified atmosphere of 5% CO_2_ at 37°C. The cells were treated with fisetin in different concentrations (10, 30, and 100 μM) for indicated time.

### Cell Viability Assay

The effect of fisetin on cell viability was assessed using the 3-(4,5-dimethylthiazol-2-yl)-2,5-diphenyl-2H-tetrazolium bromide (MTT) assay. Exponentially growing MDA-MB-231 and BT549 cells were seeded at 8 × 10^3^ cells per well in 96-well culture plates, and cultured for 12 h before treatment with a series of concentrations (10, 30, and 100 μM, respectively) of fisetin. Each concentration set 3 replicates. Blank control cells were treated with DMEM alone. After incubation for 72 h, 20 μL of 5 mg/mL MTT was added to each well and incubated for another 4 h. After that, the medium containing MTT was removed and replaced by 120 μL/well of DMSO to solubilize the blue formazan crystals. Absorbance at 490 nm was detected with an automated microplate reader (ELx800, BioTek, United States).

### Wound Healing Assay

MDA-MB-231 and BT549 cells were seeded into six-well plates in culture medium and allowed to grow to 100% confluence. A sterile toothpick was used to scrap the center of wells to create wounds. The scraped cells were washed out with PBS and the remaining cells continued to be cultured in the absence or presence of different concentrations of fisetin. After 24 h, cells were observed and the gap distance of the wound was measured. Two time points (0 and 24 h after scrap) were selected to capture the wound healing of MDA-MB-231 and BT549 cells by a light microscope (Nikon, Japan).

### Transwell Invasion Assay

The invasion of MDA-MB-231 and BT549 cells was measured using the Matrigel-coated Transwell chamber (Corning, MA, United States). Briefly, cells at a density of 2.5 × 10^5^ cells/well were seeded onto the upper chambers in a serum-free medium with or without series concentrations of fisetin, the lower chamber was filled with DMEM medium supplemented with 10% FBS. Following incubation for 24 h, the cells on top surface were scraped, and the cells on the lower surface of the membranes were fixed with 4% paraformaldehyde for 10 min and stained using 0.25% crystal violet for 15 min. The invaded cells were counted at 200× magnifications using a microscope (Nikon, Japan).

### Immunofluorescent (IF) Staining

For IF analysis, MDA-MB-231 and BT549 cells treated by series concentrations of fisetin were fixed in 4% paraformaldehyde for 10 min, permeabilized with 0.05% Triton X-100 for 10 min at room temperature and incubated with primary antibodies overnight at 4°C. The cells were then incubated with fluorescein-conjugated secondary antibodies for 1 h at room temperature in darkness. At last DAPI was added to counterstain the nucleus. The primary tumor tissues were stained in the same way, except that tissues were immersed in blocking solution containing 1% BSA in PBS after permeabilized. Images of cells and tissues were captured using fluorescence microscope (Nikon, Japan).

### Western Blot Analysis

MDA-MB-231 and BT549 cells were cultured in absence or presence of series concentrations of fisetin for 24 h, then harvested and lysed in RIPA buffer to isolate the total protein. After incubation for 30 min on ice, lysates were centrifugated at 12,000 *g* for 5 min and the supernatants were collected and stored at −80°C. The protein concentration was detected by using the BCA kit according to the manufacturer’s instructions. Equal amounts of denatured proteins (30 μg) were electrophoresed on 10% SDS gel and subsequently transferred to polyvinylidene fluoride (PVDF) membranes. After being blocked in Tris-Buffered Saline containing 0.1% Tween-20 (TBST) for 1 h, the membranes were incubated with an optimal dilution of the desired primary monoclonal antibodies at 4°C overnight. After washing with TBST for three times, the membranes were incubated with an optimal dilution of the appropriate secondary antibodies conjugated with horseradish peroxidase (HRP) for 2 h at the room temperature. At last use the enhanced chemiluminescent system and X-ray to make the membranes visualization.

### Quantitative Reverse Transcription-Polymerase Chain Reaction (qRT-PCR)

Briefly, total RNA was extracted from cells following the manufacturer’s instruction of the RNA extraction kit. Total RNA (1 μl) was reverse transcribed to complementary DNA (cDNA) by using the PrimeScript RT reagent Kit. At last qPCR was performed by using the PCR kit following the instruction. The specific primer sequences we used were as following: E-cadherin: 5′-TCCTGGGCAGAGTGAATTTTGAAGA-3′ (forward), 5′-AAACGGAGGCCTGATGGGG-3′ (reverse); Claudin: 5′-CCTCCTGG GAGTGATAGCAAT-3′ (forward), 5′-GGCAACTAAAATAGCCAGACCT-3′(reverse); Vimentin: 5′-TACAGGAAGCTGCTGGAAGG-3′ (forward), 5′-ACCAGAGGGAGTGAATCCAG-3′ (reverse); N-Cadherin: 5′-AGCCAACCTTAACTGAGGAGT-3′ (forward), 5′-GGCAAGTTGATTGGAGGGATG-3′ (reverse); Snail: 5′-TCGGAAGCCTAACTACAGCGA-3′ (forward), 5′-AGATGAGCATTGGCAGCGAG-3′ (reverse); Slug: 5′-GGGGAGAAGCCTTTTTCTTG-3′ (forward), 5′-TCCTCATGTTTGTGCAGGAG-3′(reverse); PTEN: 5′-TGGATTCGACTTAGACTTGACCT-3′ (forward), 5′-GCGGTGTCATAATGTCTCTCAG-3′ (reverse); GAPDH: 5′-TGTTGCCATCAATGACCCCTT-3′ (forward), 5′-CTCCACGACGTACTCAGCG-3′ (reverse). GAPDH primers were used as internal control and equal loading.

### Transient Transfection of siRNA

Briefly, cells were seeded into 24-well plates and incubated to about 50% confluence, then MDA-MB-231 cells were transfected with Ad-siPTEN or Ad-RFP, respectively. After 48 h transfection, the levels of PTEN protein and mRNA were analyzed by Western blotting and qRT-PCR, respectively.

### Immunohistochemistry (IHC) Staining

Tissue sections were cut into 5 μm thick after fixed with 4% paraformaldehyde and embedded. Then, tissues were deparaffinized, rehydrated, antigen repaired, and blocked with 5% goat serum. Endogenous peroxidases were quenched by incubating with hydrogen peroxide, followed by incubating with primary antibodies at 4°C overnight and HRP-conjugated second antibodies sequentially. Finally, the sections were visualized with DAB staining and imaged.

### Xenograft Model

Four to five-week-old female nude mice were obtained from the Animal Ethics Committee of Chongqing Medical University and housed in specific pathogen free (SPF) laboratory environment. The protocol was reviewed and approved by the Animal Ethics Committee of Chongqing Medical University. We chose MDA-MB-231 cells for determining the effects of fisetin *in vivo*. Female nude mice were injected subcutaneously with 1 × 10^6^ MDA-MB-231 cells into bilateral gluteal regions. When the tumor reached 100 mm^3^ in volume, mice were divided randomly into sham-treated group and fisetin-treated group. The former received PBS served as control and the latter received 100 mg/kg fisetin in an intraperitoneal injection way every 3 days. Tumor sizes were measured every 3 days. After 4 weeks, all mice were sacrificed under anesthesia, and the tumors and lungs were excised, weighted, and counted for the tumor nodules on the lung. The fixed tumor tissues were further analyzed.

### Statistical Analysis

All experiments were repeated at least three times. Data was expressed as mean ± SD. Student’s *t*-test and one-way ANOVA analysis were used to analyze the variances between groups. *P* < 0.05 was considered statistically significant.

## Results

### Fisetin Suppressed the Proliferation, Migration and Invasion of TNBC Cells *in Vitro*

To examine the anticancer effect of fisetin on TNBC cells, we treated the highly aggressive MDA-MB-231 cells and BT549 cells with fisetin in different concentration (10, 30, and 100 μM, respectively). Firstly, we assessed its proliferative activities through MTT assay at 72 h. It can be observed that fisetin inhibited the cancer cells proliferation in a dose-dependent way (**Figure 1B**). Then we used wound-healing and transwell assay to determine whether fisetin had the potential to inhibit breast cancer cells migration and invasion. Fisetin could concentration-dependently slow down the wound healing process comparing to the control group (**Figures 1C,D**), and a similar effect was observed in transwell assay, the invaded cells were decreased in fisetin-treated groups when compared with the control group (**Figures 1E,F**). All these results suggested that fisetin had the anticancer capability through influencing the proliferation, migration and invasion of TNBC cells.

### Fisetin Reversed EMT in TNBC Cells *in Vitro*

**FIGURE 2:**
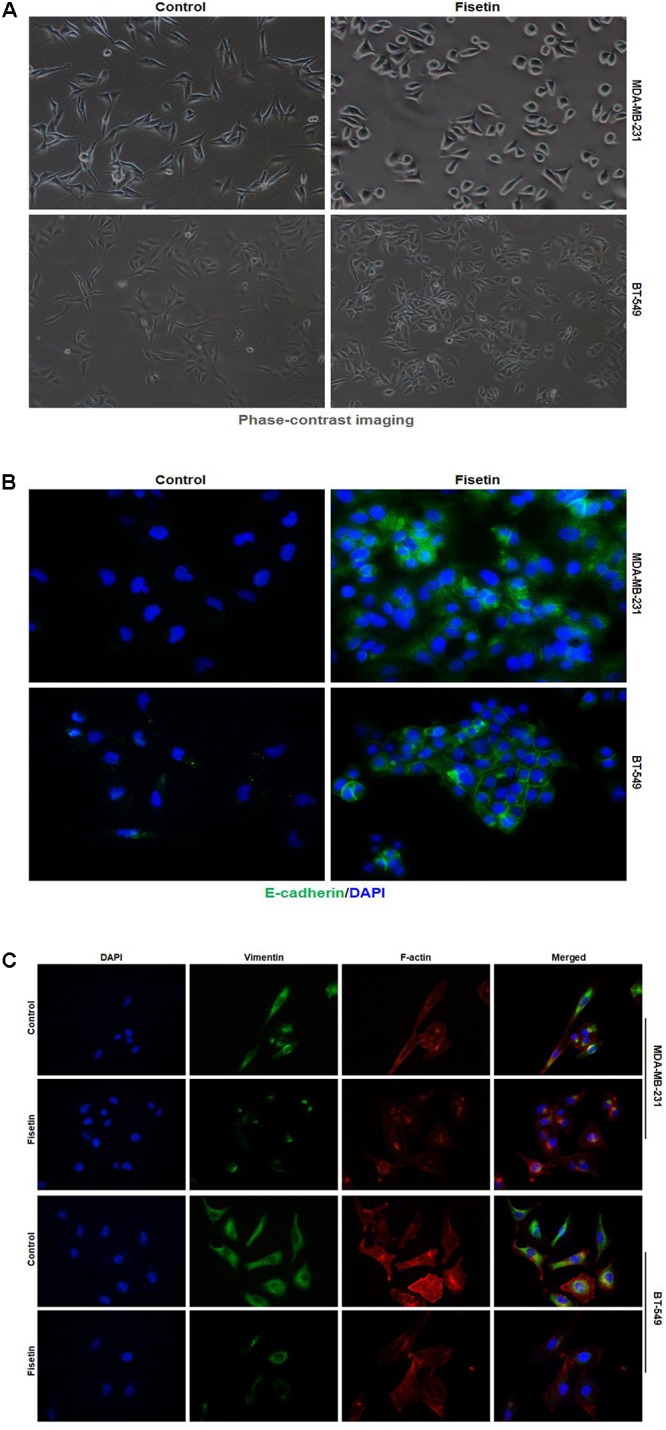
Fisetin reverses EMT in TNBC cells *in vitro*. TNBC cell lines MDA-MB-231 and BT549 were treated with vehicle or fisetin for 24 h. **(A)** The morphology of the cells treated with vehicle or 30 μM fisetin was observed by phase-contrast microscopy. **(B)** E-cadherin and **(C)** Vimentin and F-actin expression were evaluated by immunofluorescence in TNBC cells treated by vehicle or 30 μM fisetin. The cells pretreated with vehicle or various concentrations of fisetin (10, 30, and 100 μM, respectively) were subjected to western blot for the indicated proteins **(D)** and qRT-PCR for the indicated mRNA **(E)**. The results are shown as the mean ± SD of three experiments, ^∗^*P* < 0.05, ^∗∗^*P* < 0.01 compared with control.

Epithelial to mesenchymal transition is an important process related to the metastasis of tumor cells. For the inhibitory function of fisetin on invasion and migration in MDA-MB-231 and BT549 cells, we explored whether fisetin might achieve it through regulating the EMT process. Therefore, to determine the relationship between fisetin and EMT, we used 10, 30, and 100 μM of fisetin to treat MDA-MB-231 and BT549 cells, followed by exploring the shift of cell morphology and evaluating the expression of EMT markers. The two TNBC cell lines presented a long spindle mesenchymal-like feature, while treated with fisetin, cancer cells were changed into oval epithelial-like type (**Figure 2A**). The immunofluorescence results showed a visible up-regulation of E-cadherin and down-regulation of Vimentin at the concentration of 30 μM fisetin, and the cytoskeletal protein F-actin in the cytoplasm was remolded (**Figures 2B,C**), suggesting that our hypothesis might be right, in which fisetin had the potential to suppress EMT. So furthermore, we quantitatively detected the expression of EMT markers by Western blot and qRT-PCR. These two assays demonstrated that both at the protein and mRNA levels, the epithelial markers E-cadherin and Claudin were up-regulated but the mesenchymal markers N-cadherin and Vimentin changed in the opposite way, at the same time, the EMT related transcription factor Snail but not Slug was down-regulated (**Figures 2D,E**).

### Fisetin Suppressed PI3K-Akt-GSK-3β Signal Pathway but Upregulated PTEN Expression *in Vitro*

**FIGURE 3:**
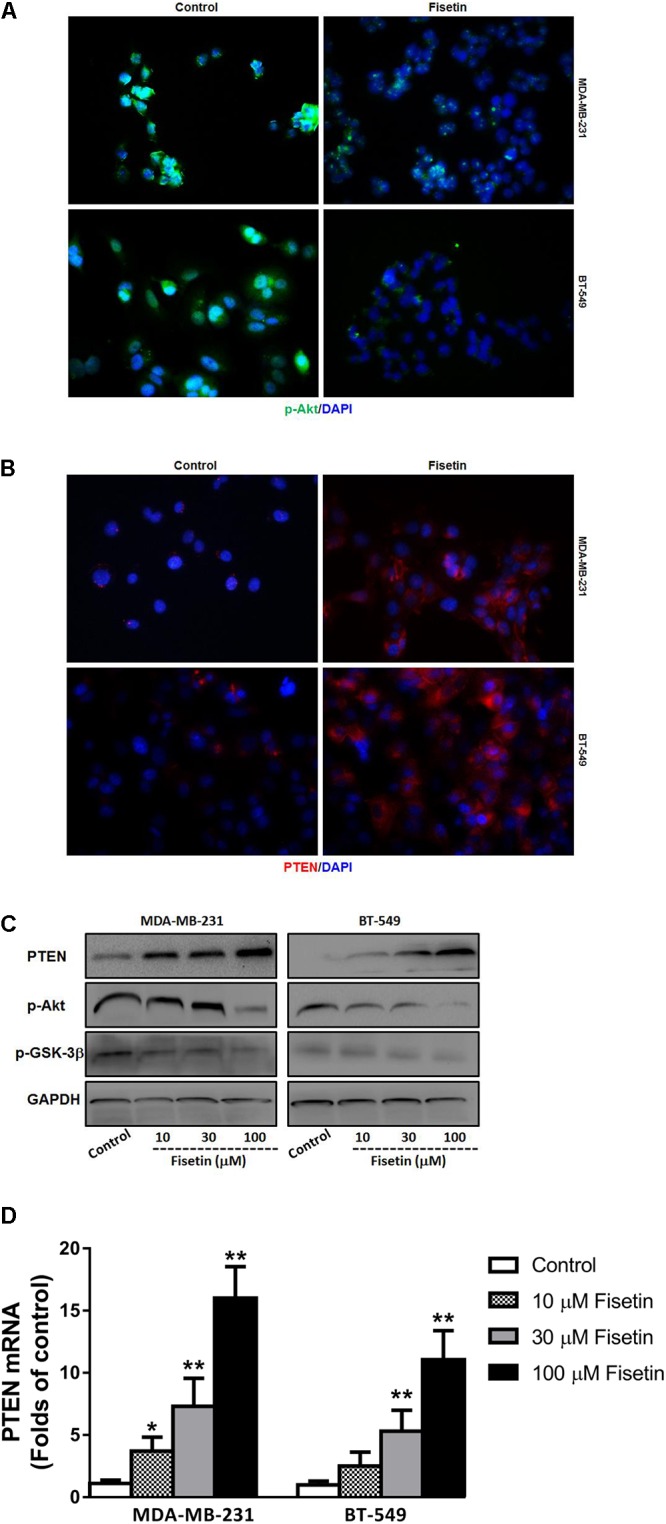
Fisetin suppresses PI3K-Akt-GSK-3β signal pathway but upregulates PTEN expression *in vitro*. TNBC cell lines MDA-MB-231 and BT549 were treated with vehicle or 30 μM fisetin for immunofluorescence assay and with vehicle or various concentrations of fisetin (10, 30, and 100 μM) for western blot and qRT-PCR. **(A)** The expression of p-AKT was evaluated by immunofluorescence. **(B)** The expression of PTEN was evaluated by immunofluorescence. **(C)** The expression of PTEN protein as well as p-AKT and p-GSK-3β was determined by western blot. **(D)** The expression of PTEN mRNA was determined by qRT-PCR. The results are shown as the mean ± SD of three experiments, ^∗^*P* < 0.05, ^∗∗^*P* < 0.01.

As PI3K/Akt/GSK-3β signaling pathway plays an important role in promoting the process of EMT and mediating the metastasis of cancer, and PTEN can act as a phosphatase to dephosphorylate Akt, so we detected the expression of the key members in this PTEN-Akt-GSK-3 signaling molecules. Immunofluorescent staining results showed that the expression of p-Akt was downregulated, but PTEN was upregulated after being treated with 30 μM fisetin comparing to the control group (**Figures 3A,B**). Moreover, the result of western blot in **Figure 3C** showed that comparing to the control group, both in the two cell lines, the protein level of PTEN was upregulated by fisetin in a dose-dependent way, while the expression of p-Akt and p-GSK-3β was decreased in the same way. Consonantly, using the qRT-PCR method to assay the expression of PTEN mRNA, we found that fisetin dose-dependently unregulated the expression of PTEN mRNA in two TNBC cell lines (**Figure 3D**).

### Silencing of PTEN Abrogated the Effects of Fisetin on TNBC Cells Proliferation and Metastasis as Well as EMT

**FIGURE 4:**
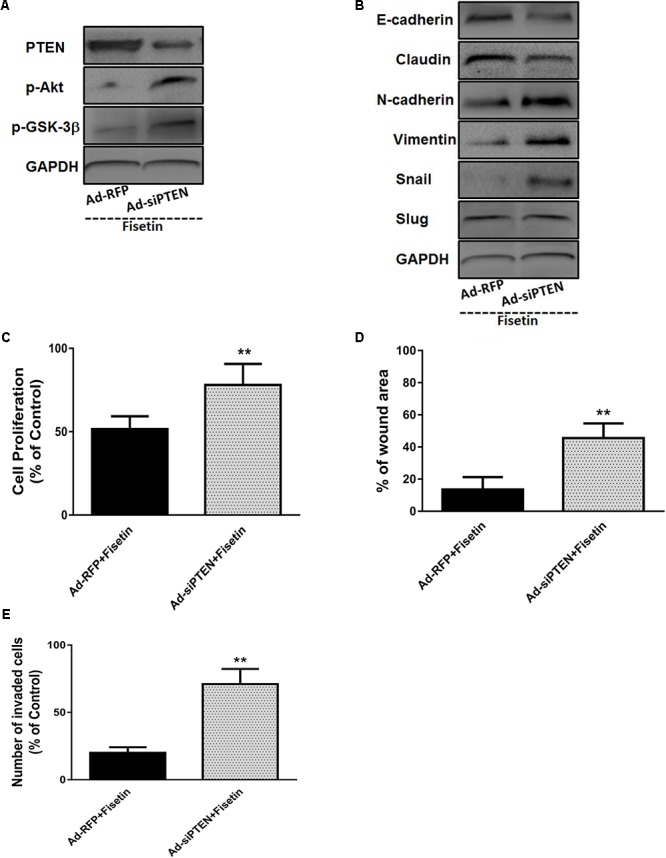
Silencing of PTEN abrogates the effects of fisetin on TNBC cells proliferation and metastasis as well as EMT. TNBC cell line MDA-MB-231 cells were transfected with Ad-RFP or Ad-siPTEN, and subsequently treated with fisetin (100 μM). **(A)** The expression of PTEN and p-AKT and p-GSK-3β protein was determined by western blot. **(B)** EMT molecule markers were determined by western blot. **(C)** The cell proliferation was determined by MTT assay. **(D)** The cell migration was determined by wound-healing assay. **(E)** The cell invasion was determined by transwell invasion assay. The results are shown as the mean ± SD of three experiments, ^∗^*P* < 0.05, ^∗∗^*P* < 0.01 compared with control.

To evaluate whether the antitumor effects of fisetin is mainly correlated with the upregulation of PTEN which can inhibit Akt signaling, the expression of PTEN was silenced with Ad-si PTEN in MDA-MB-231 cells. As shown in **Figure 4A**, the decrease of PTEN and increase of p-Akt and p-GSK-3β were observed in Ad-si PTEN transfected MDA-MB-231 cells treated by fisetin (100 μM) when compared with Ad-RFP control group. Moreover, using western blot method, we found that those beneficial changes of fisetin on EMT markers E-cadherin, Claudin, N-Cadherin, Vimentin and related transcription factor Snail, were also abrogated by PTEN silence (**Figure 4B**). Intriguingly, anti-proliferation (**Figure 4C**), anti-migration (**Figure 4D**), and anti-invasion (**Figure 4E**) effects of 100 μM fisetin was counteracted by the silence of PTEN.

### Fisetin Inhibited the Growth and Metastasis of TNBC *in Vivo*

**FIGURE 5:**
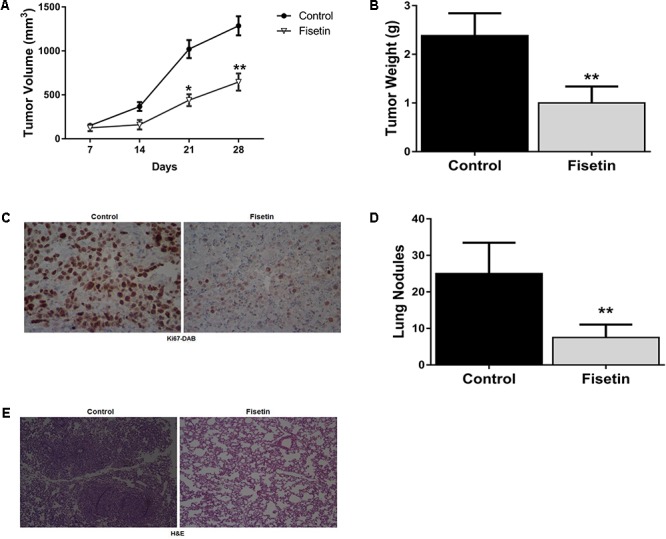
Fisetin inhibits the growth and metastasis of TNBC *in vivo*. Xenograft breast cancer model was established by subcutaneous injection of MDA-MB-231 cells in the presence or absence of fisetin (100 mg/kg). **(A)** Tumor growth curve was recorded. **(B)** Tumor weight was measured. **(C)** The expression of Ki67 in the primary tumor tissues was evaluated by immunohistochemistry staining. **(D)** Metastatic tumor nodules on the surface of lungs were counted. **(E)** Representative images of HE staining in the metastatic nodules of lungs. The results are shown as the mean ± SD of six experiments, ^∗^*P* < 0.05, ^∗∗^*P* < 0.01 compared with control.

To evaluate the anti-proliferation and anti-metastasis potential of fisetin *in vivo*, we used the xenograft metastasis tumor model bearing MDA-MB-231 cells. Results indicated that the primary tumors isolated from fisetin-feeded mice exhibited a dramatic decrease in tumor growth volume (**Figure 5A**) and weight (**Figure 5B**) comparing with the control group. IHC staining of Ki-67 on the primary tumor tissues also clarified that fisetin could significantly reduce the area of cancer nests and decrease the proliferation ability of breast cancer cells (**Figure 5C**). Moreover, we found the number of the prominent metastatic nodules on the surface of lungs were less in fisetin-treated mice than control mice (**Figure 5D**). HE staining of lung tissue sections isolated from mice received orthotopic transplantation also showed that fisetin dramatically suppressed TNBC cells metastases to the lung (**Figure 5E**).

### Fisetin Inhibited PTEN-Akt-GSK-3β Signaling Pathway and Reversed EMT *in Vivo*

**FIGURE 6:**
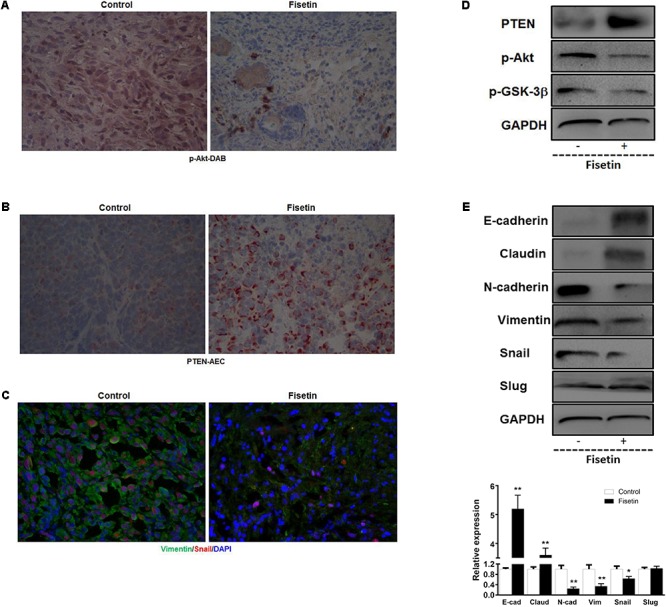
Fisetin inhibits PI3K/AKT/GSK-3β signaling pathway and reverses EMT *in vivo*. Xenograft tumor metastasis was established by subcutaneous injection of MDA-MB-231 cells in the presence or absence of fisetin (100 mg/kg). **(A)** AKT activation was evaluated by immunohistochemistry staining. **(B)** Representative images of PTEN stained section of tumor tissues isolated from nude mice bearing breast cancer. **(C)** Vimentin and Snail in primary tumor tissues were examined by immunofluorescence assay. **(D)** PTEN and p-Akt and p-GSK-3β protein in the primary tumor tissues was examined by western blot. **(E)** EMT molecule markers were determined by western blot. The results are shown as the mean ± SD of six experiments, ^∗^*P* < 0.05, ^∗∗^*P* < 0.01 compared with control.

To confirm the pathophysiological relevance of our *in vitro* observations, we examined the related signal molecules in tumor tissues of xenografted model implanted with MDA-MB-231 cells. In agreement with the *in vitro* results, IHC analysis of the xenograft primary tumor tissues revealed an apparent down-regulation of p-Akt (**Figure 6A**) and upregulation of PTEN (**Figure 6B**) in fisetin-treated group. In addition, immunofluorescent analysis showed that mesenchymal marker molecule Vimentin and transcription factor Snail were significantly inhibited by fisetin compared with xenografted model mice (**Figure 6C**). Coincident with the results above, western blot analysis showed that PTEN as well as the epithelial markers E-cadherin and Claudin were increased but p-Akt and p-GSK3β and the mesenchymal markers N-cadherin, Vimentin with the EMT transcription factor Snail were decreased in the orthotopic tumor tissues of mice after fisetin treatment (**Figures 6D,E**).

## Discussion

Nowadays, breast cancer is no longer considered as a single disease but a systemic diseases, and the complete diagnosis dependent on the histopathology report of the tumor which assess the presence or absence of the hormone receptors for estrogen (ER), progesterone (PR), and the human epidermal growth factor receptor-2 (HER-2) (Shaikh et al., 2016). Therefore, it is a heterogeneous disease which can be divided into many different subtypes including luminal A and B, HER2-enriched, basal-like, and normal breast-like (Tao et al., 2014). These factors are the basis for us to choose therapy strategy, because some of these factors have been associated with the survival rate of patients and their clinical outcome after treatment (Hernandez-Aya and Gonzalez-Angulo, 2013). However, in all of the molecular subtypes, triple-negative breast cancer (TNBC) which is characterized by a loss of ER, PR and HER-2 is the most tough situation and the total number of the patients approximately accounts for 10–20% of all breast cancer patients that is not a small percentage that can be ignored (Boyle, 2012). Patients with TNBC usually have a poor prognosis and high rates of metastasis because of the lack of targets for endocrinotherapy and targeted therapy (Li et al., 2016), chemotherapy is the major treatment they can take after surgery. However, the generation of chemoresistance leads the anti-cancer treatment into a dilemma. Fisetin, a structurally distinct chemical substance that belongs to the flavonoid group of polyphenols, has been reported to exert the beneficial effects on anti-cancer (Murtaza et al., 2009; Szliszka et al., 2011; Chou et al., 2013). In breast cancer, there are several studies confirm that fisetin can induce cell cycle arrest, caspase-dependent apoptosis, inhibit autophagy, and enhance cytotoxicity of chemotherapeutic agents (Yang et al., 2012; Smith et al., 2016). As indicated in this study, fisetin effectively inhibited the proliferation, migration and invasion of MDA-MB-231 and BT549 cells, and suppressed the growth and lung metastasis of breast cancer in the xenograft model bearing MDA-MB-231 cells, indicating that fisetin may be used as an effective drug for treatment of metastatic TNBC patients.

Triple negative breast cancer, a subtype of breast cancer characterized by its highly aggressive and metastatic features, has been proved to have close relationship with EMT. The promotion of EMT programs apparently drives TNBC metastasis partly by eliciting cytoskeleton rebuilding from apical-basolateral polarities characteristic of epithelial cells to the synthesis of actin stress fibers characteristic of mesenchymal cells (Taylor et al., 2013), during which, the morphology, motility and polarity all perform a dramatic shift, and at the molecule level, the epithelial markers E-cadherin and Claudin are lost and the mesenchymal markers Vimentin and N-cadherin are gained (Ombrato and Malanchi, 2014). When the EMT process was inhibited by miRNAs or the silence of the transcription factors, metastasis of TNBC was reversed at the same time (Rhodes et al., 2014; Lv et al., 2016). As have been proved that fisetin had the certain potential on inhibition of migration and invasion in TNBC, we supposed whether the anti-metastasis function was associated with EMT. So in the present study, we detected the EMT related biomarkers in MDA-MB-231 and BT549 cells, and found that after being treated with fisetin, both of the two cell lines acquired epithelial features and lost mesenchymal phenotype, which was coincident with the previous reports describing that fisetin-inhibited EMT in nasopharyngeal carcinoma cells and prostate cancer cells, respectively (Khan et al., 2014; Li et al., 2014). *In vivo* experiments, we also got the same results in primary tumor and metastatic lung tissues. Furthermore, we detected the expression of two major EMT-related transcription factors Snail and Slug and found that fisetin also dose-dependently inhibited the expression of Snail. All these data demonstrated that the inhibitory effect of fisetin on metastasis of TNBC is closely connected with reversion of EMT.

Cancer metastasis and EMT are complicated processes regulated by numerous chiasmatic signaling pathways such as Wnt/β-catenin, hedgehog, and PI3K/Akt signaling pathways. Among these signal pathways, PI3K/Akt pathway is one of the major regulators. In breast cancer, a number of researches have reported that once PI3K/Akt pathway is inhibited, cancer metastasis will be suppressed at the same time (Ren et al., 2014; Chang et al., 2016). Akt can be activated by the lipid kinase PI3K through generating the second messenger PIP3 (phosphatidylinositol-3,4,5- triphosphate). GSK-3β is an important downstream molecule of Akt, which has close relationship with cellular proliferation, migration, apoptosis, cell cycle and glucose regulation (Ali et al., 2001; Umezawa et al., 2016). Activation of the PI3K-Akt-GSK-3β signaling pathway makes the downstream transcription factor Snail more stable to repress the expression of gene CDH 1 encoding E-cadherin, promoting EMT process (Lamouille et al., 2014). PTEN (phosphatase and tensin homolog deleted on chromosome 10) is a well-known tumor suppressing gene and the deletion or mutation of PTEN is usually involved in tumor development (Keniry and Parsons, 2008). PTEN emerges the anti-cancer effects partly because it can negatively regulate PI3K/Akt pathway through counteracting the activity of PI3Ks via dephosphorylating PIP3 into PIP2 (phosphatidylinositol- 4,5-bisphosphate) (Chalhoub and Baker, 2009). PTEN level and function are regulated transcriptionally, post-transcriptionally, and post-translationally. On transcriptional level, it can be positively regulated by a wealth of transcription factors, such as early growth response protein 1 (EGFR-1), peroxisome proliferator activated receptor γ (PPAR-γ), and tumor protein 53 (Tp53), which can directly bind to PTEN promoter region, while other transcription factors show the negative regulation of PTEN in several cancer models, such as mitogen activated protein kinase kinase-4 (MKK4), transforming growth factor β (TGF-β), and the polycomb group (PcG) protein BMI1 (Bermudez Brito et al., 2015). In breast cancer, PTEN expression also can be suppressed by promoter methylation (Garcia et al., 2004). Histone modifications is another epigenetic mechanism by which PTEN expression can be suppressed (Mirmohammadsadegh et al., 2006). On post-transcriptionally level, miRNAs play an important role on the regulation of PTEN expression. Dong et al. (2014) have demonstrated that the upregulated miR-21 reduced the PTEN expression in TNBC. In this study, using MDA-MB-231 and BT549 cells, we firstly demonstrated the expression of PTEN could be dose-dependently up-regulated by fisetin at mRNA and protein levels, along with the reduction of p-Akt and p-GSK-3β activation. And *in vivo*, with the breast cancer xenograft model bearing MDA-MB-231 cells, we further found that fisetin also apparently up-regulated the expression of PTEN and inhibited p-Akt in primary tumor tissues. According to this arresting discovery, coupled with the subtle relationship between PTEN-Akt-GSK-3β signaling and EMT and tumor metastasis, we speculated that the anti-metastasis effect of fisetin on breast cancer was mediated by PTEN-Akt-GSK-3β signaling pathway, and in which, PTEN was the most important kernel molecule. To prove this hypothesis, we used siRNA to make PTEN silenced in MDA-MB-231 cells before fisetin intervention, and found that the restraint effects of fisetin on metastasis and EMT was counteracted, implying that up-regulation of PTEN expression is the key point to the inhibitory function of fisetin on tumor metastasis and EMT. But how PTEN was upregulated by fisetin should be further researched. It may through suppressing the negative transcription factors or miRNAs, or promoting the positive transcription factors, or interfering in the epigenetic modification way.

## Conclusion

Our present study authenticated that the natural flavonoid fisetin manifested a potential agonistic activity on metastasis of TNBC, which might be the results from reversing of EMT by inhibition of PTEN-Akt-GSK-3β signaling pathway. These findings provided supporting evidence to make fisetin to be recognized as a novel potential therapeutic agent for the treatment of TNBC patients with metastatic breast cancer.

## Author Contributions

All authors listed have contributed to the work and approved it for publication. JW, GK, and HL conceived and designed the experiments. JL, XG, RJ, DL, HL, and TZ performed the experiments. XG, JW, GK, and JL analyzed the data. JL, JW, and HL wrote the paper.

## Conflict of Interest Statement

The authors declare that the research was conducted in the absence of any commercial or financial relationships that could be construed as a potential conflict of interest.
